# Human DLG1 and SCRIB Are Distinctly Regulated Independently of HPV-16 during the Progression of Oropharyngeal Squamous Cell Carcinomas: A Preliminary Analysis

**DOI:** 10.3390/cancers13174461

**Published:** 2021-09-04

**Authors:** Lucija Lulić, Antonia Jakovčević, Luka Manojlović, Emil Dediol, Lawrence Banks, Vjekoslav Tomaić

**Affiliations:** 1 Division of Molecular Medicine, Ruđer Bošković Institute, Bijenička cesta 54, 10000 Zagreb, Croatia; Lucija.Lulic@irb.hr; 2 Clinical Department of Pathology and Cytology, University Hospital Center Zagreb, 10000 Zagreb, Croatia; antonia.jakovcevic@gmail.com; 3 Department of Pathology and Cytology, University Hospital Dubrava, 10000 Zagreb, Croatia; luka.manojlovic@kbd.hr; 4 Department of Maxillofacial Surgery, University Hospital Dubrava, 10000 Zagreb, Croatia; emildediol@yahoo.com; 5 Tumour Virology Laboratory, International Centre for Genetic Engineering and Biotechnology, Padriciano 99, 34149 Trieste, Italy; banks@icgeb.org

**Keywords:** HNSCC, OPSCC, HPV, E6, p16, DLG1, SCRIB

## Abstract

**Simple Summary:**

The process of HPV-mediated oncogenesis in HNSCCs is not fully understood. DLG1 and SCRIB protein expression levels and localization changes were evaluated in a number of HPV16-positive and HPV-negative OPSCCs and seem to be associated with malignant transformation. Moreover, loss of SCRIB expression inversely correlates with higher grade tumors, and this is much more evident in the presence of HPV16 E6. This could serve as a potential marker in predicting development of OPSCCs.

**Abstract:**

The major causative agents of head and neck squamous cell carcinomas (HNSCCs) are either environmental factors, such as tobacco and alcohol consumption, or infection with oncogenic human papillomaviruses (HPVs). An important aspect of HPV-induced oncogenesis is the targeting by the E6 oncoprotein of PDZ domain-containing substrates for proteasomal destruction. Tumor suppressors DLG1 and SCRIB are two of the principal PDZ domain-containing E6 targets. Both have been shown to play critical roles in the regulation of cell growth and polarity and in maintaining the structural integrity of the epithelia. We investigated how modifications in the cellular localization and protein expression of DLG1 and SCRIB in HPV16-positive and HPV-negative histologic oropharyngeal squamous cell carcinomas (OPSCC) might reflect disease progression. HPV presence was determined by p16 staining and HPV genotyping. Whilst DLG1 expression levels did not differ markedly between HPV-negative and HPV16-positive OPSCCs, it appeared to be relocated from cell–cell contacts to the cytoplasm in most samples, regardless of HPV16 positivity. This indicates that alterations in DLG1 distribution could contribute to malignant progression in OPSCCs. Interestingly, SCRIB was also relocated from cell–cell contacts to the cytoplasm in the tumor samples in comparison with normal tissue, regardless of HPV16 status, but in addition there was an obvious reduction in SCRIB expression in higher grade tumors. Strikingly, loss of SCRIB was even more pronounced in HPV16-positive OPSCCs. These alterations in SCRIB levels may contribute to transformation and loss of tissue architecture in the process of carcinogenesis and could potentially serve as markers in the development of OPSCCs.

## 1. Introduction

Head and neck squamous cell carcinomas (HNSCCs) are the most common form of head and neck cancers and are the sixth most common cancer worldwide, with more than 930,000 new cases and more than 460,000 deaths in 2020 [1,2]. HNSCCs are roughly divided into two groups based on human papillomavirus (HPV) status: HPV-negative HNSCCs are caused by environmental risks such as smoking or chewing tobacco, and heavy alcohol consumption; while a proportion of HNSCCs are also caused by infection with mucosotropic HPVs, predominantly type HPV16 [3]. Surprisingly, HPV-positive and HPV-negative oropharyngeal squamous cell carcinomas (OPSCCs) seem to be two different entities of the same disease; they show differences regarding their biology, therapeutic response, and even survival prognosis, since HPV-positive OPSCCs can result in better survival rates than HPV-negative. However, this also depends on multiple factors [4].

HPVs are small non-enveloped double-stranded DNA viruses associated with almost 100% of cervical cancers, 70% of anogenital cancers, 40–60% of OPSCCs and 5% of non-OPSCCs [2,5]. HPV carcinogenesis requires the combined action of two major viral oncoproteins, E6 and E7, which interact with various cellular proteins [6], including two of the most important tumor suppressors, p53 and pRb, respectively [7,8]. Furthermore, E6 oncoproteins from oncogenic HPVs contain a Class I PDZ (PSD-95/DLG1/ZO-1) binding motif (PBM) at their carboxy termini, allowing them to interact with a number of cellular PDZ domain-containing proteins. PBM–PDZ domain interactions seem to be important for the viral life cycle, since PBM mutations contribute to a lower number of episomal HPV genomes and reduced cell growth rate. Likewise, these interactions play pivotal roles in cellular transformation and cancer development in transgenic mice models [9,10]. Two of the tumor suppressor proteins from the Scribble complex, DLG1 and SCRIB, are amongst the most well-characterized PDZ domain-containing substrates targeted by oncogenic E6 proteins for proteasome-mediated degradation, both in vitro and in vivo [11,12]. DLG1 is a member of the MAGUK (membrane associated guanylate kinase homologues) protein family and is expressed in a variety of cells, including epithelia, where it localizes in the membrane, making DLG1 essential for regulation of polarity and cellular growth in response to cell–cell contact [13]. In addition, SCRIB is required for maintenance of an epithelial phenotype at low cell densities [14]. Therefore, loss or delocalization of these proteins during cancer progression leads to the loss of cell polarity and invasiveness that is observed during metastasis [15]. More importantly, these interactions seem to play critical roles in the later stages of development of HPV-induced malignancies [9,10].

The process from the initial infection to cancer development in the head and neck (HN) region is still poorly understood. It appears to be much shorter than in the cervix/uterus where it usually takes 12–15 years before a persistent HPV infection may result in development of cervical carcinoma [16]. Unfortunately, HN malignancies are mostly diagnosed at an advanced stage, due to the difficulty of early detection [17]. Currently, immunohistochemical (IHC) detection of the tumor suppressor p16 is widely used as a surrogate marker for transcriptionally-active high-risk (HR) HPVs, since the E7 oncoprotein induces its overexpression [18]. However, 10–20% of p16-positive/HPV-negative OPSCCs [19] and p16-negative/HPV-positive OPSCCs have been reported, showing that there is no exclusive link between p16 overexpression, HPV infection, and cancer progression [20], and hence p16 expression should be used as a diagnostic indicator only if supported by HPV-genotyping. Likewise, around 80% of cervical cancers are caused by two types, HPV16 and HPV18 [12], while HPV16 accounts for 90–95% of all HPV-induced OPSCCs [5]. This suggests potential differences in the interaction profiles of known E6/E7 cellular substrates at these two anatomical sites, which could be responsible for the heterogeneity in the malignant transformation. The results of several studies investigating DLG1 function in cervical cancer development suggest that its expression and localization varies in HPV-associated lesions, probably due to the combined action of the viral oncoproteins E6 and E7 [21]. Moreover, it seems that DLG1 may be important for the progression of low-grade cervical intraepithelial lesions (LSILs), since it was shown to be upregulated and preferentially cytoplasmic in all LSILs that progressed to high-grade squamous intraepithelial lesions (HSIL), but very low or almost undetectable in invasive carcinoma [22]. Similarly, it seems that the upregulation of Scribble in tumors may contribute to a translocation of other proteins of the family away from their functional location, causing aberrant cell polarity and proliferation [23]. Since the majority of HPV-positive OPSCCs result from HPV16 infections and SCRIB protein is known to be a preferred HPV16 E6 PDZ domain-containing cellular target [24], we examined possible differences in the expression levels and localization of DLG1 and SCRIB in HPV16-positive and HPV-negative formalin-fixed paraffin embedded (FFPE) OPSCC samples, with the aim of better understanding the process of HPV-induced carcinogenesis in the HN area. Here, we show that DLG1 and SCRIB are predominantly relocated from cellular membranes to the cytoplasm in tumors, regardless of HPV status. However, interestingly, SCRIB expression appears to be downregulated in HPV-positive and in poorly differentiated HPV-negative cancers graded 3/3, suggesting its potential as a biomarker. Although the loss of SCRIB is observed in tumors regardless of HPV-status, it seems to be much more substantial in the presence of HPV16.

## 2. Materials and Methods

### 2.1. Tissue Samples

The study was conducted on FFPE HNSCC samples (*n* = 65), HPV16-positive oropharyngeal cancer (*n* = 21), HPV-negative oropharyngeal cancer (*n* = 36), and healthy tonsillar tissue (*n* = 8). The tissue samples were obtained from the archives of the Department of Pathology and Cytology, University Hospital Dubrava (ethical permit no. BEP-55 48/2-2016) and the Department of Pathology and Cytology University Hospital Centre, Zagreb, Croatia (ethical permit no. 02/21 AG, Class 8.1-21/15-2). All the tissue samples were previously fixed in 10% buffered formalin and embedded in paraffin. 

### 2.2. DNA Detection and Genotyping

From each FFPE sample, five to seven slices (10 µm) were used for DNA isolation. DNA was isolated using a commercial kit (NucleoSpin^®^ DNA FFPE XS, Macherey–Nagel), according to the manufacturer’s instructions. The concentration of the isolated DNA was measured using NanoPhotometer^®^ N60 (Implen GmbH), and the quality was validated by PCR using primers generating 99bp long beta-actin fragments [25].

For HPV DNA detection, PCR was performed using short primers suitable for FFPE tissue samples GP5/6 (~142bp) and SPF 10 (~65bp) [26,27]. PCR amplification was performed as previously described [28]. The PCR products (10 μL) were run on 3% agarose gels (Sigma Aldrich). A sample was considered to be HPV-positive if either the GP5/6 or SPF10 PCR was positive. In addition, for the detection of HPV16 E6, a supplementary primer pair, generating a shorter DNA sequence (98bp), was used [29]. HPV16 E6 DNA was amplified using the following thermocycling steps: initial denaturation at 95 °C for 10 min; 40 cycles of 95 °C for 1 min, 54 °C for 1 min and 72 °C for 2 min; with a final elongation at 72 °C for 7 min.

### 2.3. Antibodies

Endogenously expressed DLG1 and SCRIB were detected in FFPE tissue sections using mouse monoclonal antibodies SAP 97 (2D11) and Scrib (C-6) (Santa Cruz Biotechnology, Inc.) at 1:20 dilution. Biotinylated secondary antibodies were provided in commercial kits CINtec^®^ Histology kit system (Roche Holding AG) and EnVision^®^ + Dual Link System-HRP (Agilent).

### 2.4. Immunohistochemistry

Slices (7 µm) of the FFPE tissue sections were mounted on pretreated glass slides, deparaffinized in xylene substitute BioClear (Biognost, Zagreb, Croatia), and rehydrated using a graded ethanol series (100%, 95% and 70%). The expression of p16 was analyzed using the CINtec^®^ Histology kit system (Roche Holding AG, Basel, Switzerland) and compared with the HPV status of the corresponding tissue. Endogenous expression of DLG1 and SCRIB was detected using EnVision^®^ + Dual Link System-HRP (Agilent, Santa Clara, CA, USA) according to the manufacturer’s instructions. Negative controls for each sample were processed in the same way, except that the primary antibody was replaced with the negative control solution provided (CINtec^®^ Histology kit system), in p16 staining or antibody diluent (10% FBS, 1% BSA, 0.3% Triton-X TBS). The intensity of DLG1 and SCRIB immunostaining was graded and scored by three independent pathologists as follows: 0 (no staining), 1+ (low intensity), 2+ (medium intensity), and 3+ (strong intensity).

### 2.5. Statistical Analysis

Descriptive statistics were used to evaluate differences in DLG1 and SCRIB expression and localization in HPV-positive and HPV-negative OPSCCs.

A Kruskal–Wallis test was used to evaluate possible significant differences between the designated groups, HPV- OPSCCs grades 1–3 and HPV16+ OPSCCs with Dunn’s multiple comparison posttest. The ratio of DLG1- or SCRIB-positive staining in tumor cells was evaluated by three independent pathologists.

Pearson’s χ2 test was used for evaluation of any significant differences in DLG1 or SCRIB localization between designated groups: HPV- OPSCCs grades 1–3 and HPV16+ OPSCCs. All statistical analyses were performed using GraphPad Prism 5 software.

## 3. Results

### 3.1. The Expression of p16

A total of 66 OPSCC and 8 normal, non-cancerous and HPV-negative tonsillar tissue samples were collected. DNA was isolated, and PCR detection of HPV16 E6 was performed, as described in Materials and Methods. Of 66 OPSCC samples, 21 were classified as HPV16-positive and 36 as HPV-negative OPSCC. The remaining nine samples were excluded, as they were either inconclusive due to insufficient DNA or were HPV-positive but not HPV16-positive.

**Figure 1:**
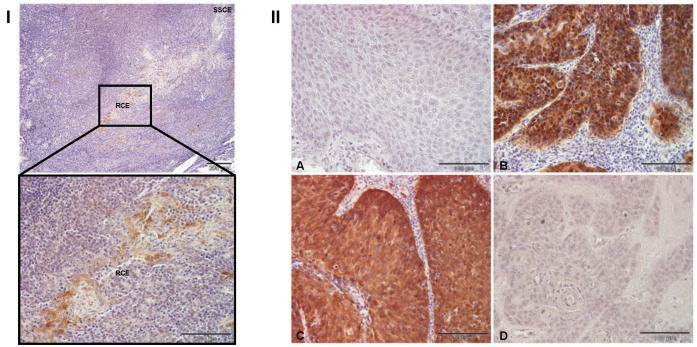
Immunohistochemical analysis of p16 expression. Paraffin-embedded biopsies were immunostained for p16, counterstained with hematoxylin, and visualized by a light microscope. Representative images: (**I**) Positive p16 immunoreactivity is seen in the reticulated crypt epithelium (RCE) but not in the superficial squamous cell epithelium (SSCE). (**II**) A. No p16 immunoreactivity was detected in the majority of HPV-negative OPSCCs. B. p16 immunoreactivity was seen in a few HPV-negative OPSCCs. C. p16 immunoreactivity was detected in the majority of HPV16-positive OPSCCs. D. No p16 immunoreactivity was seen in a few HPV16+ OPSCCs. Scale bars = 200 μm and 100 μm (**I**); 100 μm (**II**A–D).

The expression of p16 protein was investigated using IHC in all samples, including normal tonsillar tissue (Figure 1). As expected, p16 positivity was not an exclusive biomarker of HPV16 positivity. According to the obtained data, 18/21 (85.71%) of HPV16-positive OPSCCs and 3/36 (8.33%) of HPV-negative OPSCCs were positive for p16, which correlates well with the results of previous studies [19,20]. Representative pictures of every combination of p16 and HPV positivity are shown in Figure 1II. In normal tonsillar tissue (Figure 1I), we did not detect the typical p16 staining, as seen in the tumors; instead, we observed specific staining only in cryptal epithelium, as has been described previously [30].

### 3.2. DLG1 and SCRIB Protein Expression Patterns in Non-Cancerous Tonsillar Tissue Samples

**Figure 2:**
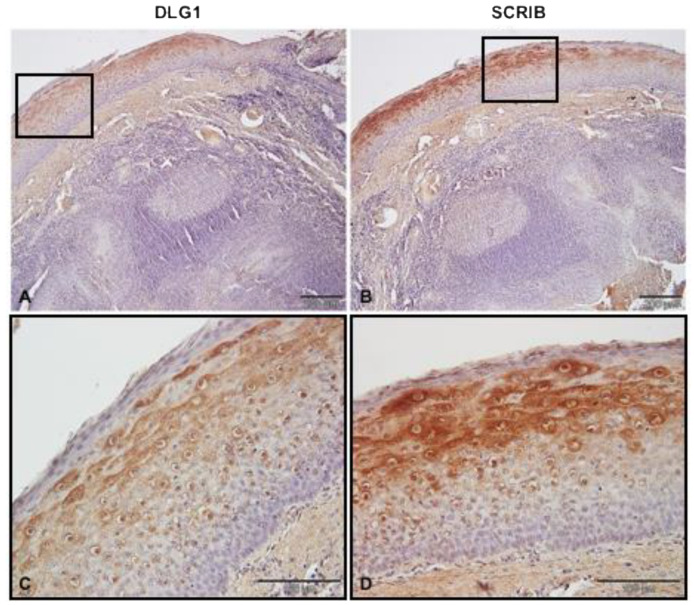
DLG1 and SCRIB expression in cancer-free normal tonsillar tissues. Paraffin-embedded biopsies were immunostained with anti-DLG1 and anti-SCRIB antibodies, counter stained with hematoxylin, and visualized by a light microscope. Representative images: (**A**) Positive cytoplasmic and membranous DLG1 immunoreactivity in squamous cell epithelium. (**B**) Positive cytoplasmic and membranous SCRIB immunoreactivity in squamous cell epithelium. (**C**,**D**) Enlargements of (**A**) and (**B**), respectively. Scale bars = 200 μm (**A**,**B**); 100 μm (**C**,**D**).

Before investigating any potential differences in the expression levels and localization of SCRIB and DLG1 during malignant progression in the oropharynx, we investigated their patterns of expression in eight non-cancerous tonsillar tissue samples, using IHC (Figure 2). The specificity and working dilutions of the antibodies were assessed by Western blot analysis and immunocytochemistry on three different cell lines—normal oral keratinocytes, tonsillar keratinocytes, and human foreskin keratinocytes (data not shown). We detected protein expression of both SCRIB and DLG1 in 8/8 non-cancerous tonsillar tissue samples. The protein expression patterns were similar in all samples; both SCRIB and DLG1 were expressed mostly in the intermediate layer of stratified squamous epithelia, while they were almost absent in the basal and superficial layers. SCRIB and DLG1 were predominantly expressed in the cytoplasm of cells in the upper parts of the intermediate layer.

### 3.3. Analysis of DLG1 and SCRIB Protein Expression and Localization in HPV-Negative OPSCC Samples

**Figure 3:**
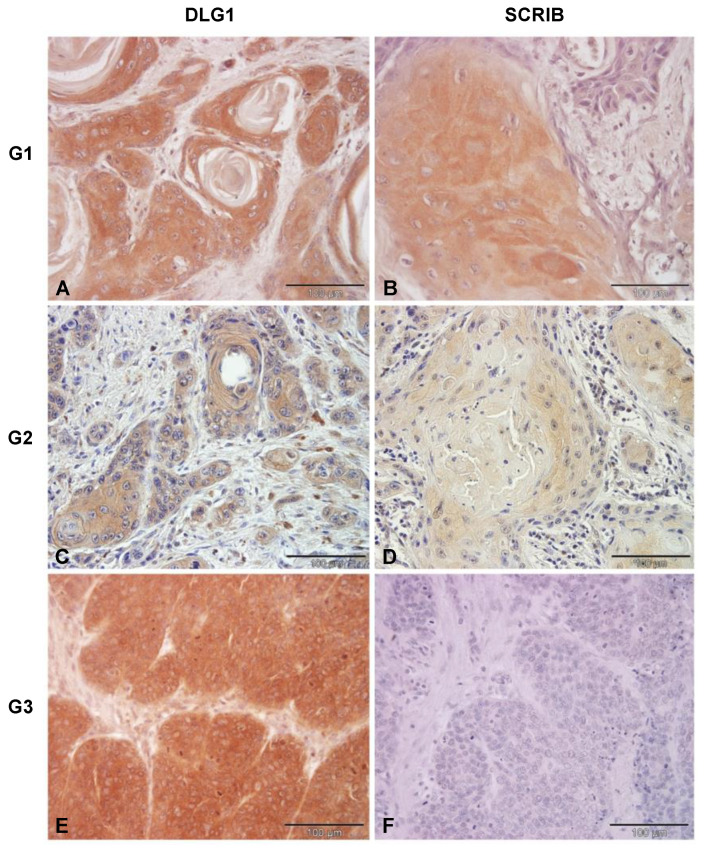
DLG1 and SCRIB protein expression levels in HPV-negative OPSCCs of various grades (G). Paraffin-embedded biopsies were immunostained with anti-DLG1 and anti-SCRIB antibodies, counterstained with hematoxylin, and visualized by a light microscope. Representative images: G1. (**A**) Positive cytoplasmic and membrane DLG1 immunoreactivity. (**B**) Positive cytoplasmic SCRIB immunoreactivity. G2. (**C**) Positive cytoplasmic and membrane DLG1 immunoreactivity. (**D**) Positive cytoplasmic SCRIB immunoreactivity. G3. (**E**) Positive DLG1 immunoreactivity. (**F**) Negative SCRIB immunoreactivity. Scale bars = 100 μm.

We started by analyzing 36 HPV-negative OPSCC samples whose HPV negativity was confirmed by genotyping. These samples were divided into three groups as classified by a pathologist; G1: well differentiated (7/36), G2: moderately differentiated (10/36), and G3: Poorly differentiated or undifferentiated (19/36). Representative examples of each group are shown in Figure 3. In the well differentiated OPSCCs, DLG1 protein was detected in all samples with predominantly low to middle intensity, and it was equally localized in the cytoplasm and at cell–cell contacts. We then proceeded to monitor SCRIB protein expression changes in the same tissue samples. In those, the majority of samples showed low to middle intensity, similar to that observed in the normal tonsillar tissue. SCRIB was predominantly localized in the cytoplasm of cancer cells, while we detected SCRIB both in the cytoplasm and at cell–cell contacts in only one sample. In moderately differentiated HPV–negative OPSCCs, DLG1 was absent in 2/10 of OPSCCs and was expressed at a low intensity in one sample. The remaining samples demonstrated either a medium-intensity (5/10) of DLG1-positive staining or a high-intensity (2/10), as observed in one of the non-cancerous tonsils. Similarly, DLG1 protein was evenly localized in the cytoplasm and at cell–cell contacts. No SCRIB was detected in 2/10 samples, 4/10 showed only a low-intensity SCRIB staining, 3/10 showed a medium-intensity staining, and in only one sample was the SCRIB staining comparable to that seen in healthy tissue. As in the histologic samples described above, SCRIB protein was mostly localized in the cytoplasm, and in just one sample were both cytoplasmic and membranous staining detected. Most of the HPV-negative OPSCCs were classified as poorly-differentiated. In only one G3 sample was DLG1 completely absent, while the vast majority showed either a low or medium DLG1 expression signal. In most of these samples, DLG1 was localized mainly in the cytoplasm but was also detected at cell–cell contacts. In contrast, SCRIB protein was completely absent from 9/19 samples. A further 9/19 showed low-intensity SCRIB staining, and one sample had a medium-intensity SCRIB staining in a low proportion of cancerous cells. Again, SCRIB was predominantly localized in the cytoplasm, although we also observed membranous staining in two samples.

In short, only marginal changes in levels of DLG1 expression were seen in all three groups of HPV-negative OPSCCs when compared to cancer-free healthy tonsillar tissues. However, the presence of relocated, and thus functionally altered DLG1, could potentially be caused by modifications of DLG1 at the post-transcriptional and/or post-translational levels, rather than being caused by genomic mutations in this set of tumors. Although some mutations in the PDZ-2 domain of DLG1 have been found in breast cancer, there is still no clear explanation of their effects, if any, on tumor development and/or progression. However, a possible explanation is that these PDZ-2 domain mutations in DLG1 might prevent it from forming complexes with other proteins such as APC and PTEN [31,32], thus not being able to optimally perform its cellular functions. On the other hand, the loss of SCRIB seems to increase with the disease progression. We also observed delocalization, suggesting that some post-translational mutations, such as S-palmitoylation, could be involved in this process, resulting in a disruption of cell polarity and loss of SCRIB’s tumor suppressive activities [33]. In addition, some point mutations in SCRIB, like P305L in the leucine rich repeat (LRR) region, were reported to cause its relocation from the membrane to the cytoplasm in organotypic 3D MCF10A cultures, which could also be responsible for the phenotype observed in the tumor samples [34].

### 3.4. Analysis of DLG1 and SCRIB Protein Expression and Localization in HPV16+ OPSCC Samples

**Figure 4:**
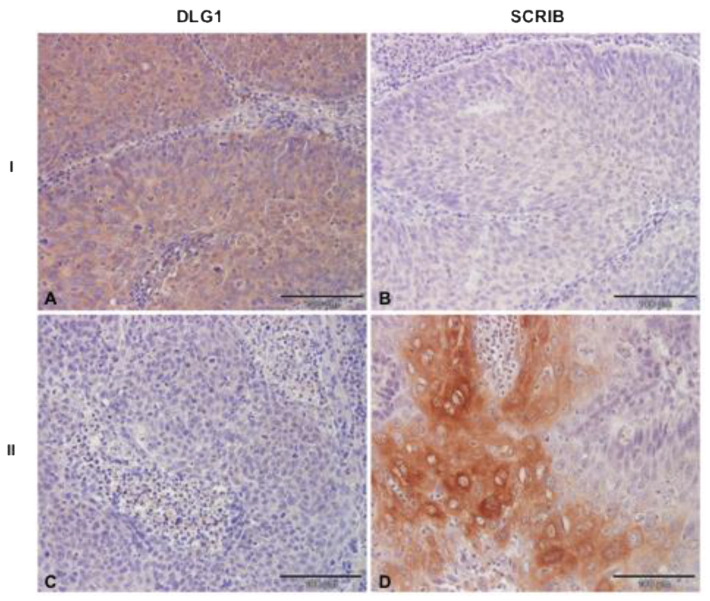
DLG1 and SCRIB protein expression levels in HPV16-positive OPSCC. Paraffin-embedded biopsies were immunostained with anti-DLG1 and anti-SCRIB antibodies, counterstained with hematoxylin, and visualized by a light microscope. Representative images: I. Pattern observed in the majority of samples: (**A**) positive cytoplasmic DLG1 immunoreactivity; (**B**) negative SCRIB immunoreactivity. II. Conflicting pattern observed in several samples: (**C**) negative DLG1 immunoreactivity; (**D**) positive SCRIB immunoreactivity. Scale bars = 100 μm.

Since cell polarity regulators SCRIB and DLG1 have been previously characterized as cellular targets of E6 oncoproteins [11], we wanted to examine potential changes in the protein expression patterns and cellular localization of these two substrates in HPV16-positive OPSCCs, using the same protocol as for the HPV-negative ones. Representative images of the analyses are shown in Figure 4. Of the samples that we analyzed, DLG1 protein was absent in only 3/21 samples, which is consistent with the previously reported preference of HPV16 E6 for targeting SCRIB over DLG1 [24]. This observation is also in agreement with the results from the HPV-negative OPSCCs, which show a similar profile of DLG1 protein expression. Of the 18/21 samples that stained positive for DLG1, 8/21 showed low-intensity cytoplasmic staining, while 9/21 samples showed medium-intensity staining, similar to that observed in normal tonsillar tissue, and only 1/21 sample showed high-intensity staining in the majority of cancer cells. In the tumors analyzed, DLG1 predominantly localized in the cytoplasm (13/21), while 5/21 showed both cytoplasmic and membrane DLG1 positivity, similar to the pattern observed in the healthy tonsillar tissue. In contrast, the vast majority of samples (17/21) showed complete ablation of SCRIB protein, as expected, since SCRIB undergoes proteasomal-mediated degradation in the presence of HPV16 E6 protein, and this E6-mediated SCRIB turnover is increased in the more malignant stages of the disease [15]. Of the remaining four samples, in 3/21, a low-intensity SCRIB staining was detected, and only 1/21 showed a higher intensity staining in a small proportion of all cancer cells.

**Figure 5:**
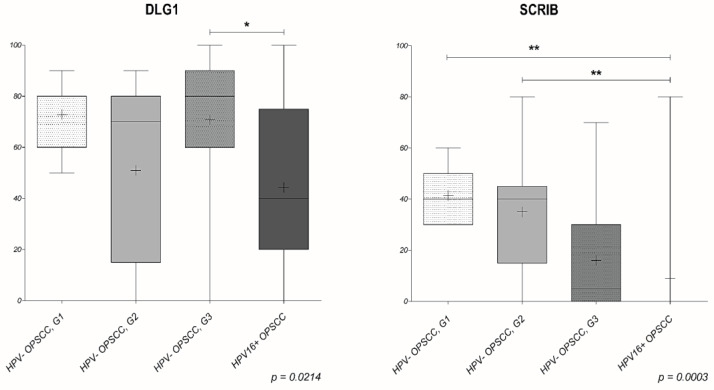
The ratio of DLG1- and SCRIB-positive staining in HPV-negative OPSCCs of various grades and HPV-positive OPSCCs. The score of DLG1 (I)- and SCRIB (II)-positive staining observed by pathologists was evaluated between HPV16-positive and HPV-negative OPSCCs grades 1–3 using the Kruskal–Wallis test with Dunn’s multiple comparison posttest. I. *p*-value (*) 0.0214, II. *p*-value (**) 0.0003.

**Figure 6:**
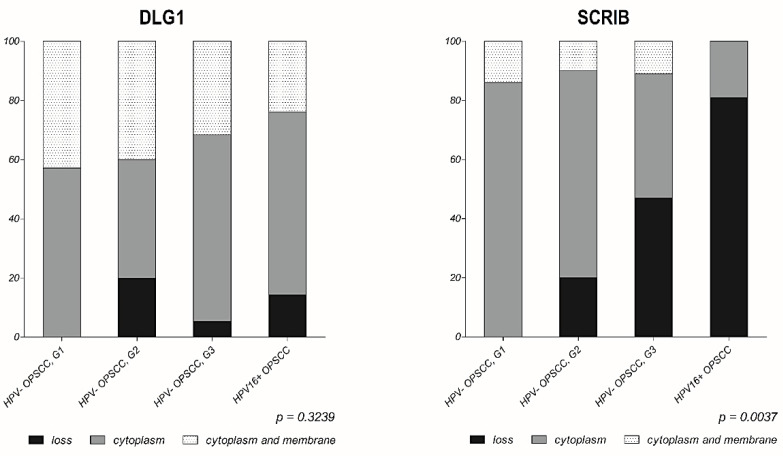
Changes in expression and localization of DLG1 and SCRIB proteins in HPV-positive OPSCCs and HPV-negative OPSCCs of various grades. Black bars show the percentage of FFPE samples with a complete loss of antigen. Light grey bars represent the percentage of samples with only cytoplasmic localization of antigen. Dark grey bars represent the percentage of samples with both cytoplasmic and membranous localization of antigen. *p*-values are 0.3239 and 0.0037 for DLG1 and SCRIB, respectively.

More interestingly, in those samples where SCRIB was still detectable, it was localized solely in the cytoplasm of cancer cells, suggesting that relocation of SCRIB from cell–cell contacts may contribute to cell transformation in these tumors. These results are in line with previous studies showing that epithelial cell differentiation [35], various genetic, post-transcriptional and post-translational modifications [34,36], and HPV infection [37,38] can contribute to changes in the cellular distribution of both SCRIB and DLG1. In addition, when comparing the percentage of SCRIB and DLG1 expression levels in different groups (Figure 5), we noticed a reduction in SCRIB levels concurrent with the progression of HPV-negative malignancies, and this is much more evident in the presence of HPV16 E6. Although we had rather small sample sizes, we observed a significant change (*p* = 0.0003, Figure 5) in SCRIB expression levels in HPV16-positive OPSCCs when compared to either well-differentiated (G1) or moderately-differentiated (G2) HPV-negative OPSCCs. However, this was not observed with DLG1; the DLG1 expression levels in HPV16-positive OPSCCs seemed to change significantly (*p* = 0.0214, Figure 5) only in comparison with the poorly-differentiated (G3) HPV-negative group, and therefore fluctuations in DLG1 protein are not likely to contribute to disease progression in the same way as SCRIB. On the other hand, the change in protein localization was greater for DLG1 than for SCRIB in both HPV-negative and HPV16-positive OPSCCs (Figure 6), again suggesting differences in the modulation of these two proteins during malignant progression, regardless of HPV16 presence.

## 4. Discussion

In the past 30 years, HPV infection has been recognized as a causative agent of HPV-positive OPSCCs, with HPV16 being responsible for almost 90% of them [2,5]. Numerous studies have characterized HPV-induced OPSCCs epidemiologically, clinically, anatomically, and biologically as a distinct disease from HPV-negative OPSCCs. Although rising in prevalence compared with HPV-negative OPSCCs, HPV-positive OPSCCs have a favorable prognosis when treated with chemotherapy, radiation, surgery, or chemoradiotherapy [39], but early detection of primary tumors and prevention of the disease progression are still needed for survival rates to improve. Currently, the guidelines for HPV detection in OPSCCs have not met the gold standard and are not strictly defined. Even though IHC staining of p16 is widely used as a surrogate marker for diagnosis of HPV-induced OPSCC, approximately 10% of HPV-negative HNCs show p16 overexpression [40]. This points to the need for other potential prognostic biomarkers that could be more accurate in predicting the development of the disease. Therefore, we decided to investigate changes in the expression patterns and localization of the two previously characterized E6 targets, DLG1 and SCRIB, in HPV-negative and -positive OPSCCs; such changes in their expression patterns having been shown to be correlated with loss of tissue architecture during malignant progression at various anatomical sites [35,37]. This would additionally contribute to a better understanding of the roles of these two tumor suppressors in the process of oncogenesis in the HN region and further elucidate their potential biomarker significance for predicting the onset of the disease in the early stages.

In our combined analysis of PCR genotyping and p16 IHC staining, we found that 3/21 (14.3%) of the total HPV16-positive samples analyzed were p16-negative when stained using the CINtec^®^ histology kit. Similarly, 3/36 (8.3%) of HPV-negative samples were p16-positive. As expected, our results reaffirmed p16-positivity in both HPV-negative and HPV-positive OPSCCs, additionally supporting the need for new biomarkers, more closely correlating with HPV positivity, and more predictive of disease development in oropharyngeal tumors. DLG1 was shown to be implicated in the process of malignant transformation, since loss of polarity is recognized as a fundamental step in carcinogenesis [41]. Furthermore, DLG1 is a known target of multiple human viral oncoproteins, including HR HPV E6, the adenoviral E4-ORF1, and the human T cell leukemia virus type 1 [31]. The fact that E6 targets DLG1 for proteasome-mediated degradation suggested that DLG1 protein perturbations in HPV-infected cervical epithelium might have prognostic value. Various groups worldwide have reported the loss of DLG1 expression in later, more malignant and invasive stages of the disease [37,38], while in LSIL, DLG1 was upregulated and relocated to the cytoplasm [22,37,38]. Taken together, these data suggest that DLG1 may play an important role in cervical cancer development. Indeed, similar observations were reported in some non-HPV-mediated cancers, indicating potential mechanistic similarities in cancer development, regardless of HPV presence; DLG1 was downregulated in moderately-differentiated colon adenocarcinoma and was almost undetectable in poorly-differentiated tumors [35,37]. Likewise, DLG1 downregulation was observed in the later stages of lung, laryngeal, breast, and hepatocellular cancers [31].

Therefore, in this study we first wanted to determine whether similar changes occur in OPSCCs and, if so, whether changes in the expression levels and/or localization of DLG1 are dependent upon HPV status. In healthy and non-infected tonsillar tissue, DLG1 was expressed both in the cytoplasm and at cell–cell contacts, mostly in the parabasal, intermediate layer of stratified squamous epithelium, but was absent from the basal and superficial layers. Although these results differ somewhat from studies of normal cervical epithelium, which found DLG1 in the basal and parabasal, but not in the superficial, layers [37,38], this DLG1 expression pattern is similar to that found in tonsillar tissues in The Human Protein Atlas [42]. Next, to highlight any potential changes in the expression levels and localization of DLG1, we analyzed a series of HPV-negative OPSCC samples, classified as well-, moderately-, or poorly-differentiated. In the well-differentiated (7/36) samples, DLG1 expression levels varied, but the localization—cytoplasmic and at cell–cell contacts—was similar to that in normal, non-infected tonsillar tissue. In moderately-differentiated OPSCCs (10/36), DLG1 expression was lost in two samples, but in the remaining, it was of a medium- or high-intensity, with cytoplasmic and membranous localization again similar to that seen in non-cancerous tonsils. Finally, in poorly-differentiated or undifferentiated OPSCC samples (19/36), DLG1 expression was lost in only one sample, while the vast majority showed either low or medium expression, with cytoplasmic and membranous localization. Our results show a slight increase in relocation of DLG1, suggesting that deregulation of normal DLG1 function is associated with disease progression; again, this supports the hypothesis of DLG1 acting both as a tumor suppressor and cell polarity regulator [31,43]. We then proceeded to investigate any potential effects in HPV16-positive OPSCC samples. In those, DLG1 expression differed significantly (*p* = 0.0214) only from that in grade 3 HPV-negative tumors. However, the slightness of changes in relocation suggests that HPV16 E6 does not markedly affect localization of DLG1, and therefore the phenotype could be a result of other, unknown, processes during cancer progression, such as the acquisition of certain mutations, like G338R, I348V, G338R/I348V, and L329R/G330R in the DLG1 PDZ-2 domain in both HPV-negative and HPV-positive tumors during the process of oncogenesis, as has been seen in breast cancer. Furthermore, these PDZ-2 domain mutations in DLG1 seem to disable its interaction with the tumor suppressors APC and PTEN, emphasizing the importance of DLG1 functions and how alterations in its protein expression and localization might contribute to the process of oncogenesis [31,32].

We then examined the same set of samples for any changes in the expression and localization of SCRIB, which is the preferred PDZ-domain containing target of HPV16 E6 [24]. Unlike DLG1, SCRIB belongs to the LRR protein family whose LRR region is fully sufficient for normal functioning in some biological processes. However, most of the known interacting partners of SCRIB associate with it via PDZ domains that seem to be the key sites for molecular networks in both epithelial cells and neurons [44]. Previous studies reported a dynamic localization of SCRIB in embryos, indicating that localization is crucial for proper functioning of SCRIB. Like DLG1, SCRIB is a key player in cell polarity regulation and is a tumor suppressor, since loss of SCRIB leads to cancerous cellular overgrowth [45]. Moreover, SCRIB has been characterized as a critical player for epithelial cell migration, in response to extracellular signals during wound healing [43]. Although it was previously reported that SCRIB was universally overexpressed in cells of various tumors including colon, liver, prostate, uterus, thyroid, lung, bladder, breast, ovary, and stomach [46], some studies have reported a down-regulation and cytoplasmic localization of SCRIB in colon, endometrial, breast, and cervical cancers [43]. Likewise, low expression of SCRIB in fibroblasts seems to be associated with the invasiveness of lung cancer cells [47].

In healthy and non-infected FFPE tonsillar tissues, we observed SCRIB expression both in the cytoplasm and at cell–cell contacts; like DLG1, it was mostly in the parabasal layer of the stratified squamous epithelium and was absent from the basal and superficial layers. This differs from The Human Protein Atlas, where SCRIB is reported to be expressed in the basal and intermediate layers of squamous epithelia but is absent from the superficial layer. The lack of basal layer SCRIB expression in these samples could be the result of inflammatory processes prior to tonsillar removal or to potential age differences between the patients. In the HPV-negative OPSCCs, SCRIB was expressed at low to medium levels in almost all the well- and moderately-differentiated samples; only one sample showed SCRIB levels that were similar to those seen in normal tonsillar tissues. SCRIB was predominantly localized in the cytoplasm of cancerous cells and absent from cell–cell contact areas. However, in moderately-differentiated HPV-negative OPSCC, we observed a complete loss of SCRIB protein in 2/10 samples. There was a greater loss of SCRIB protein in poorly-differentiated tissue samples. This is consistent with studies on breast cancer cells and on mutant transgenic mice, showing that cytoplasmic SCRIB promotes cancer development by affecting subcellular localization of PTEN and activation of the Akt/mTOR/S6 kinase signaling pathway [44,48]. Similarly, in 3D culture of MCF-10A cells, SCRIB knockdown or its delocalization from cell–cell contacts will cooperate with activated oncogenes, such as Myc, to drive epithelial cell transformation [49]. Since HPV-positive and poorly differentiated HPV-negative OPSCCs share morphological similarities, we expected to see similar results between these two sample groups. As expected, in the majority of HPV-positive OPSCCs, we observed a complete loss of SCRIB protein; in a few samples SCRIB was detected, but at low levels and exclusively in the cytoplasm of cancer cells. Therefore, we did not observe any significant changes between poorly-differentiated HPV-negative and HPV16-positive OPSCCs. However, when comparing lower graded HPV-negative tumors (well and moderately differentiated) with HPV16-positive ones, we observed significant changes in protein expression. Furthermore, significant changes were also ascertained between groups when SCRIB localization was examined. These results confirm the fact that the relocation of SCRIB is likely to be an important event in promoting malignant transformation [48]. It is possible that relocation of SCRIB promotes loss of interactions with its known cellular substrates and establishes a new interactome, causing modulations in functions of the novel interacting partners. Likewise, it is likely that SCRIB has dual functionality under certain circumstances, and that mutations or E6 push the equilibrium in the direction of growth promotion rather than inhibition. Even though the complete delocalization or loss of SCRIB protein was not an exclusive result of HPV16 infection in the HN area, we observed that the overall loss of SCRIB expression is significantly greater in HPV16-positive OPSCCs than in HPV-negative, which is in line with previous studies and highlights the impact of HPV16 E6 on the process of oncogenesis [37,38].

This is the first study to analyze the cause-and-effect relationship of DLG1 and SCRIB protein expression levels in a panel of HPV-negative and HPV-positive histologic oropharyngeal cancer samples. Importantly, we show a marked, differentiation-dependent reduction in SCRIB levels in the HPV-negative tumor samples, especially in grade 3 tumors, which show similarities with HPV16-positive OPSCC (Figure 5 and Figure 6). No such changes were seen in DLG1 levels, suggesting that it does not contribute to the disease progression. It is possible that all the E6 present in the samples preferentially interacted with SCRIB, or possibly with some other PDZ-domain substrates, rather than with DLG1, resulting in DLG1 levels very similar to those in grade 3 HPV-negative OPSCCs. Likewise, DLG1 loss-of-function is likely to be caused by either point mutations leading to DLG1 delocalization, or by other post-transcriptional or post-translational modifications, rather than being the direct effect of HPV 16 [32]. Indeed, we noticed a relocation of DLG1 to the cytoplasm in both HPV16-positive and HPV-negative OPSCCs, and this was more evident for DLG1 than for SCRIB. Taken together, the results from this study support the hypothesis that certain fluctuations in expression and localization of SCRIB protein are likely to correlate with higher grades of OPSCCs, and that is even more evident in the presence of HPV 16 E6. These results suggest that SCRIB could be a potential biomarker in the development of OPSCCs. However, further investigations on larger cohorts are necessary to better understand the molecular mechanisms involved in the regulation of DLG1 and SCRIB expression and how this contributes to OPSCC oncogenesis.

## 5. Conclusions

The findings of this study provide novel insights into alterations in DLG1 and SCRIB protein expression levels in a subset of HPV-positive and HPV-negative OPSCCs. We observed an increase in cellular relocation of DLG1 to the cytoplasm in tumor samples, regardless of HPV-presence, implying that deregulation of DLG1 normal function is likely to be linked with disease development. However, there was an obvious significant down-regulation in SCRIB expression levels, and this was more pronounced in poorly differentiated higher grade tumors. Interestingly, this effect was even more evident in HPV 16 E6-positive tumors. Therefore, these results imply that loss of SCRIB could serve as a potential late-stage marker in oropharyngeal carcinogenesis.

## Data Availability

The data that support the findings of this study are available on reasonable request from the corresponding author. The data are not publicly available due to privacy.

## Abbreviations

APC: adenomatous polyposis coli; DLG1, discs large homolog 1; FFPE, formalin-fixed paraffin embedded; HN, head and neck; HNSCC, head and neck squamous cell carcinoma; HPV, human papillomavirus; HR, high risk; HSIL, high-grade squamous intraepithelial lesion; IHC, immunohistochemistry; LRR, leucine rich repeats; LSIL, low-grade squamous intraepithelial lesion; MAGUK, membrane associated guanylate kinase homologues; MCF10A, non-malignant breast epithelial cell line; OPSCC, oropharyngeal squamous cell carcinoma; PBM, PDZ binding motif; PCR, polymerase chain reaction; PDZ, PSD-95/DLG1/ZO-1; PTEN, Phosphatase and tensin homologue deleted on chromosome 10; SCRIB, Scribble homolog.
